# The Sexual Politics of Anti-Trafficking Discourse

**DOI:** 10.1007/s10691-020-09447-x

**Published:** 2021-02-01

**Authors:** Prabha Kotiswaran

**Affiliations:** Dickson Poon School of Law, King’s College London, Somerset House East Wing, Strand, WC2R 2LS UK

**Keywords:** Criminal law, Sex work, Sociology, Trafficking, Palermo Protocol

## Abstract

20 years since the negotiation of the Palermo Protocol on Trafficking in 2000, the anti-trafficking field has gone from an early, almost exclusive preoccupation with sex work to addressing extreme exploitation in a range of labour sectors. While this might suggest a reduced focus on the nature of the work performed and a greater focus on the conditions under which it is performed, in reality, anti-trafficking discourse remains in the grip of polarised positions on sex work even as the carceral effects of anti-trafficking law become evident and the Swedish model of criminalising the purchase of sexual services spreads. In this article, I demonstrate how despite the recent discursive shifts to ‘modern slavery’ and ‘forced labour’, the anti-trafficking transnational legal order itself reinforces, rather than diffuses cultures of sex work exceptionalism. The growing international sex workers’ movement has offered resistance, yet a closer look at the movement and the widespread support that it has garnered for decriminalisation from international organisations, while valuable, helps reveal the greatest cost yet of anti-trafficking discourse, namely, the inability of the sex workers’ movement to produce a sophisticated theory of regulation to reduce levels of exploitation within sex work, one which is commensurate with the informality and heterogeneity of sex markets the world over. Finally, to the extent that neoabolitionist projects derive legitimacy from interventions abroad, especially in the global South, I chronicle the edifice on which it rests in one such context, namely India, to demonstrate how countries in the global South are not merely conduits for the global North’s preoccupation with moral gentrification through neo-abolitionism, but rather, that the circuits of global governmentality while influential, are highly contingent, thus producing opportunities for creative forms of mobilisation by sex workers.

## Introduction

It is 2020, almost 20 years since the 2000 United Nations (UN) Protocol to Prevent, Suppress and Punish Trafficking in Persons, Especially Women and Children (UN 2000a), supplementing the UN Convention against Transnational Organised Crime 2000 (UN 2000b), was negotiated in Palermo in December 2000. The anti-trafficking field has gone from an early, almost exclusive preoccupation of trafficking with sex work in the 1990s (Andrijasevic 2007) to addressing extreme exploitation in the production of commodities (Hodal et al. 2014; Richardson 2016; Anti-Slavery International 2016), in manufacturing, e.g. garment factories in India (Clean Clothes Campaign 2020), mining sites in Ghana (Human Rights Watch 2015), service provision e.g., nail salons and car washes in the UK (Kelly 2018) and domestic work in Lebanon (De Stone and Suber 2019).^1^ This would suggest a reduced focus on the nature of the work performed and a greater focus on its conditions. However 20 years on, anti-trafficking discourse remains in the grip of the myth of the innocent girl duped into prostitution against her will; a myth that continues to colonise our imagination and interpellate us no matter where we go—at airports, shops and sports events and through primetime TV shows, Hollywood movies and the mainstream media (Hill 2016; Sanford et al. 2016; Austin and Farrell 2017; Sardina 2019; Boecking et al. 2019).

Indeed, at a recent event to mark the end of Maria Grazia Giammarinaro’s term as UN Special Rapporteur on Trafficking in Persons, Lou de Baca, head of the US State Department’s Office to Monitor and Combat Trafficking in Persons during the Obama Administration admitted that the US’s attempt in 2000 to bring its 13th amendment^2^ jurisprudence to bear on the prostitution and sexual slavery of women and girls through the Protocol had misfired.^3^ Sex trafficking, he claimed, had “eaten the entire field” of trafficking and had chipped away at the 3Ps that the Protocol sought to promote, namely, prevention, protection and prosecution by unduly focusing on prosecution leaving us with a security paradigm. He called for a balance whereby law enforcement could prevent trafficking while minimising the collateral damage to already marginalised communities.^4^

In 2018 Maneka Gandhi, India’s Minister of Women and Child Development attempted such a balance. In an op-ed published soon after the passage of an anti-trafficking bill in 2018 in the lower house of Parliament, she wrote:

Firstly, there is an apprehension that the bill will criminalise voluntary sex work. This is completely false. On the contrary, the bill provides safeguards to voluntary sex workers against persecution and prosecution, while giving them the option to approach the magistrate for long term institutional, psychological, social and economic support if she wishes to discontinue. I urge those representing the rights of sex workers to recognize the value of this choice in the lives of the people they work so hard to defend. (Gandhi 2018)

Such a concession would have been unimaginable 20 years ago when many countries ratifying the Protocol would have treated any form of sex work as trafficking per se (GAATW 2007, 13, 17–23; Chuang 2010, 2; Wijers 2015). The minister’s statement may signal a shift in anti-trafficking discourse on sex work or may simply be a red herring. Nevertheless, it offers us an occasion to take stock of the sexual politics of anti-trafficking discourse and to tally what has changed and what has remained the same in the global North, but also in the global South. Feminists earlier spoke of a sex panic and unlikely alliances with the Christian Right (Chuang 2010, 2, 19–24) but now visualise deeper shifts such as the rightward move of radical feminists and the leftward move of evangelical Christian organisations (Bernstein 2007). Sex workers earlier emphasised the forced-free distinction within the Protocol and sex worker agency (Doezema 2005) but now look at the impact of raids, rescues and rehabilitation (Sangram 2018). To take stock, I start by offering a quick snapshot of how anti-trafficking law and policy have developed at the international level, reinforcing rather than diffusing cultures of sex work exceptionalism. By sex work exceptionalism, I mean (a) the characterisation by neo-abolitionist groups (who model themselves after 18th century abolitionists of slavery, like William Wilberforce) of the sale of sexual services for money as an egregious violation of human dignity and as an exceptionally harmful activity and (b) the overwhelming association of trafficking with trafficking for sex work and with sex work itself.

In part one, I examine the relationship between anti-sex work laws and anti-trafficking laws to illustrate the laws’ productive role in entrenching sex work exceptionalism such that the distinction between trafficking and voluntary sex work cannot be operationalised. In part two, I consider the role of the international sex workers’ movement in resisting sex work exceptionalism and how its strategies reveal the greatest cost yet of anti-trafficking discourse, namely, an inability to go beyond countering anti-trafficking discourse to produce a sophisticated theory of regulation to reduce levels of exploitation within sex work, one which accounts for the informality and heterogeneity of sex markets. Finally, in part three, while recognising that anti-trafficking discourse and sex work exceptionalism derive considerable mileage by locating the problem as being ‘out there’ in the developing world, I argue against an assumption that countries in the global South are merely conduits for the global North’s preoccupation with what Nicola Mai terms moral gentrification through neo-abolitionism (Mai 2018). I interrogate the sexual politics of anti-trafficking discourse by showing through the Indian experience, how the circuits of global governmentality while influential, are highly contingent, producing opportunities for creative forms of sex worker mobilisation.

## The Anti-Trafficking Transnational Legal Order

Sexual politics has been core to the framing of anti-trafficking law, policy and discourse since the 1900s. The most recent wave of legislative activity starting in the late 1990s is built around the Protocol. Negotiated within two years “at lightning speed on the UN clock” (Lloyd and Simmons 2015, 423), the Protocol was adopted in 2000, came into force in 2003 and has been exceptionally well ratified by 178 countries to date. The period between 2000 and 2009 witnessed the heyday of sex work exceptionalism after which closer attention to labour trafficking produced the competing frames of ‘modern slavery’ and ‘forced labour’ legislated upon in turn at the national and international levels. I have discussed the emergence of the anti-trafficking transnational legal order elsewhere (Kotiswaran 2019a). Suffice it to say that despite the prolific ratification of the Protocol, the criminal law response has been “stagnating at a low level” (UNODC 2016, 50–2). The concept of trafficking has however been remarkably resilient and adaptive over the past 20 years because of its reliance on “plausible folk theory” which Terrence Halliday and co-authors claim is built not on robust empirical foundations but on face validity, a compactness of rhetorical expression, sufficient ambiguity to accommodate conflicting understandings and an affinity with extant beliefs on crime, dirty money (Halliday et al. 2019, 17) and one might add, sex.

## Sex Work Exceptionalism

The number of states that criminalised both sex and labour trafficking in domestic laws increased from 10 percent in 2000 to about 73 percent in 2013 (Lloyd and Simmons 2015, 436) to 90 percent in 2014 (UNODC 2014). Despite this, cultures of sex work exceptionalism are alive and well because the Protocol allowed countries to regulate sex work as they saw fit. Sex work exceptionalism is operationalised through national law in at least six different ways. The most obvious form is where a state (e.g. Brazil) defines trafficking in terms of sexual exploitation (Davida 2015, 162); extreme exploitation in other sectors is considered ‘slavery’ (Skrivankova 2010, 10; ILO 2009). Similarly, the US state of Alaska redefines prostitution as sex trafficking (Mac and Smith 2018, 78). A second approach is to have a general trafficking offence (as in Romania and South Korea) that demarcates women trafficked into sex work into those who discontinue sex work (legally innocent) and those that continue (guilty) (Wijers 2015, 75, 77). A third approach is where countries which are signatories to the Protocol selectively incorporate the Article 3 definition into their domestic laws by omitting the requirement for coercion or exploitation.^5^ Thus Mexico and Bulgaria have removed coercion from their definition of trafficking thus equating adult sex workers with children (Id., 75).

In most countries though, even if the domestic law defines trafficking as per Art 3 extending beyond trafficking for sex work, in practice, anti-trafficking strategies focus on women and girls trafficked into sex work (Gallagher 2016, 6). Even here, the law does not necessarily benefit victims of trafficking because the Protocol does not exempt them from prosecution for offences committed when trafficked. Thus in a US study covering 12 counties, victims were shown to have been arrested in 59 percent of all state-level cases; these were all for low level prostitution-related offences (Farrell et al. 2016, 63). EU law has protections in this regard,^6^ and the Office of the UN High Commissioner for Human Rights issued principles and guidelines in 2002 (OHCHR 2002) which call for human rights protections for victims of trafficking.^7^ However, the 2002 Guidelines are not binding and states have enacted highly unsatisfactory non-criminalisation clauses (Kotiswaran 2018). Where such clauses exist, immunity from criminal prosecution is available only for serious crimes perpetrated as a direct consequence of the coercion applied by the trafficker. The continued punishment of trafficked victims has been serious enough for the former UN Special Rapporteur on Trafficking in Persons, Maria Grazia Giammarinaro to issue a 2020 report on non-punishment (OHCHR 2020).

Consider also, initiatives such as the New York City’s Human Trafficking Intervention Courts set up in 2013 with a view ensuring harm reduction and promoting decarceration rather than the incarceration of sex workers. Instead of being sent to jail, sex workers can avail of an adjournment in contemplation of dismissal or ACD. If they complete the mandated program and are not rearrested within a period of time, typically six months, then the charge for violating anti-sex work laws is supposed to be dismissed and all records of it sealed (Cohen and Gruber 2019, 98). Yet, in interviews, judges at these courts were unwilling to support the decriminalisation of sex work, the very source of the arrests to begin with (id. 101). Similarly, a 2019 amendment to the California Evidence Code disallows evidence as to the conduct of sex work where a sex worker is a victim of a violent crime, or where she can provide testimony as a witness to such a crime.^8^ A violent crime includes sexual assault, domestic violence, extortion, sexual battery, stalking, human trafficking, and robbery. In these situations, she need not fear being prosecuted or arrested for prostitution. Note that even here, there is no immunity per se, only an evidentiary shield and sex work remains criminalised outside of these situations. In other parts of the country, even when women are trafficked into sex work, anti-sex work laws are used against them.

The fifth way in which the Protocol promotes sex work exceptionalism is by triggering the reform of anti-sex work criminal laws, particularly, the adoption of the Swedish model, criminalising demand for sex work, in several countries including South Korea (2004), Finland (2006), South Africa (2008), Iceland (2009), Norway (2009), Canada (2014), Northern Ireland (2015), Spain (2015), France (2016), the Republic of Ireland (2017) and Israel (2018). This further criminalises sex work, except in South Africa which has a prohibitionist system, so the Swedish model ironically is a minor rollback on criminalisation.^9^ A possible sixth method is as follows. India has tried to pass an anti-trafficking law that ostensibly deals with extreme exploitation in several sectors but is based on strategies imported from its anti-sex work law. Thus, a provision on the closure of a brothel is applied to other sectors threatening to produce absurdities such as the closure of a farm or a household where forced labour is used (Kotiswaran 2018).

Finally, even where anti-sex work laws are not revamped, trafficking has become a reason to use these laws to escalate the brutalisation of sex workers (GAATW 2018, 30–41), through enhanced police profiling, heightened surveillance, entrapment, repressive raids, forced detention in government facilities, persistent risk of arrest and deportation, and increased vulnerability to exploitation and violence (Amnesty International 2016b, 9–16). Thus, the anti-trafficking transnational legal order is structured such that despite a ‘secular’ definition that covers sectors beyond sex work, it is deeply implicated in producing, maintaining and expanding sex work exceptionalism in the ways detailed above.

## The Rise of the International Sex Workers’ Movement

Sex workers have long been sceptical of the Protocol. Chronicling its negotiation in the late 1990s, Jo Doezema demonstrated how deploying the forced/voluntary divide was meant to resist a discourse of sex workers as deviants, but that it had been “co-opted and inverted, and incorporated to reinforce systems that abuse sex workers rights” (Doezema 1998, 47). The operationalisation of the Protocol in the intervening years and its instantiation of various forms of sex work exceptionalism only bear this out. Sex workers have however been at the forefront of resisting sex work exceptionalism. Over the past two decades, the international sex workers’ movement has formed alliances with the influential liberal medical establishment to contest neo-abolitionist discourse in the corridors of UN organisations, in courts, in academic peer-reviewed literature on HIV and on the streets through political struggle (Ahmed 2017). I detail the growing visibility and successes of the movement while also probing its critique of anti-trafficking discourse and its implications for redistributive projects within the sex sector. In particular, I demonstrate the high opportunity cost of engaging with the anti-trafficking complex by considering how the movement deals with issues of exploitation, regulation and hierarchies within the sex sector.

To gauge the extraordinary strides made by the international sex workers’ movement, we only need to recollect the 1980s when face to face interactions between feminists and sex workers were rare and tense. Thus Laurie Bell chronicled how feminists and sex workers met at a potluck dinner in Toronto only to remain in different rooms of the same house because sex workers smoked but feminists didn’t (Bell 1987). What began as a debate between feminists versus prostitutes/ ‘sex trade workers’/sex workers morphed into contestations between radical feminists, sex positive feminists, sex radical sex workers and materialist feminists across various iterations of the sex wars (Bazelon 2015; Cossman 2019). Fast forward 20 years, alongside the expansion of the Swedish model, the sex workers’ movement has grown from strength to strength. One only has to look at the website of the Global Network of Sex Work Projects and its global map highlighting where sex worker groups are active to understand this.

Even in the US, where most states adopt prohibitionist laws, the limited de facto immunity from prosecution in California presents a glimmer of hope. In South Africa, the Labour Appeals Court upheld a sex worker’s right against unfair dismissal although sex work is criminalised (*Kylie v CCMA*).^10^ In many South American countries that are not prohibitionist, governments and courts have recognised that sex work is work. In Mexico, constitutional challenges yielded positive results with the government recognising sex workers as non-salaried workers and acknowledging the distinction between sex work and human trafficking (GAATW 2018, 273, 294–295). Sex work is recognised as labour in Nicaragua; the Ministries of Labour in Nicaragua, Colombia and Gautemala recognise sex workers’ unions (Cabezas 2019, 49). Argentinian sex workers succeeded in having local laws that criminalised sex workers, repealed and fought for the repeal of a code in Buenos Aires that authorised fines and arrests of sex workers (id., 50). In Brazil, the Ministry of Labour and Employment recognises sex work as an occupation and buying and selling sex are not criminalised. The default position is that “not all prostitution is trafficking” and sex workers are represented on state and federal anti-trafficking councils (Blanchette and Murray 2016, 75, 79). These achievements are matched by improved mobilisation and organisational presence. In India for example, there are two national networks—the National Network of Sex Workers and the All-India Network of Sex Workers, each with dozens of members. Further, international sex workers’ networks like the Network of Sex Work Projects, now called the Global Network of Sex Work Projects, has 273 member organisations and is on par with an international NGO, complete with a strategic plan for 2016–2020, a theory of change and a monitoring and evaluation framework (NSWP 2020).

Testament to the strength of the international sex workers’ movement is the increasingly strong support for decriminalisation that we see today. Numerous international organisations have called for decriminalisation, many of them UN entities.^11^ Then there are civil society organisations supporting decriminalisation, including, the Open Society Foundation, the Global Alliance against Traffic in Women (GAATW), Human Rights Watch, Lambda Legal Defense and Education Fund, American Civil Liberties Union, Freedom Network US and Lancet. This culminated in Amnesty International’s call in 2016 for the decriminalisation and depenalisation of sex work even in the face of intense pressure from neo-abolitionists. In a rigorously argued report on decriminalisation, drawing on various international human rights law instruments, Amnesty International (AI) stated that:

Criminalisation and penalisation have a foreseeably negative impact on a range of human rights. These include the right to life, liberty, autonomy and security of person; the right to equality and non-discrimination; the right to be free from torture or cruel, inhuman or degrading treatment or punishment; the right to privacy; to the right to the highest attainable standard of health, the right to information and education; the right to freedom of opinion and expression; the right to adequate housing; the right to just and favourable conditions of work; the right to family life and to found a family; and the right to remedy for human rights abuses. (Amnesty International 2016a, 10)

The Amnesty Report holistically maps the radiating effects of criminalisation on sex workers’ lives, whether it is violence perpetrated by the state or by industry or societal stakeholders. The report focuses on sex workers’ civil and political rights, but acknowledges their socio-economic rights to social security in case they do not want to rely on sex work. It expands the policy vocabulary on sex work by introducing *de jure* and de facto prohibition to cover not only criminalisation on the books but also penalisation under administrative and municipal laws in the form of fines, detention for rehabilitation, deportation, loss of child custody and loss of social benefits. Hence, decriminalisation is not enough, depenalisation is essential. Further, states should not impose blanket criminalisation conflating trafficking and sex work. The Amnesty policy is thus an extraordinary victory for sex workers who can marshal its policy and evidence base in domestic constitutional litigation.

Upon a close reading, however, the AI policy and explanatory notes have a thin understanding of coercion, consent and exploitation. Consent is defined as a voluntary and ongoing agreement to engage in a particular sexual activity in the absence of force, deception, fraud and abuse of power—a liberal feminist formulation, accompanied by a reliance on rights discourse. However, as critical legal scholars like Jane Scoular have cautioned, rights are not just about democratising power but are themselves a mode of power which we must use with great caution (Scoular 2015, 101). Indeed we can take every right that AI argues is offended by criminalisation and flip it around to argue (as neo-abolitionists have) that sex work violates these very rights of sex workers (see Geist 2016). Also, there is no critique in the Amnesty report of the anti-trafficking paradigm; instead it is the very distinction between voluntary sex work and trafficking which is coerced, that enables Amnesty’s robust claim for the decriminalisation of sex work. Recollect here the forms of sex work exceptionalism which I have demonstrated, are entrenched by anti-trafficking law itself.

These weaknesses of Amnesty’s policy also affect the international sex workers’ movement. Feminists and sex workers around the world have recently come together in a remarkable effort to comprehensively document the negative effects of anti-trafficking policy. In the space of just a few years, we have a six-country empirical study of sex work commissioned by GAATW (GAATW 2018), two special issues of the Anti-Trafficking Review on sex work (2016, 2019), at least three special issues of the Open Democracy blog Beyond Trafficking and Slavery on sex work (2016, 2017, 2020) and a Red Umbrella Fund supported study in 2016 focusing on 13 sex worker organisations in nine countries and two regional networks (Vollbehr 2016). The 2016 AI Report was also based on an empirical study of sex work in four countries (Amnesty International 2016c). Sex workers’ groups have also produced reports: the 2010 Report, Human Rights, Sex Work and the Challenge of Trafficking by the x:talk project in the UK (x:talk, 2010), the 2012 Report *Hit and Run* by Bangkok-based sex workers’ group Empower (Empower 2012) and the 2018 report *Raided* by Indian group Sangram (Sangram 2018).

These reports while crucial, are formulaic in demonstrating the negative consequences of anti-trafficking policies for sex workers and in insisting on the distinction between trafficking and sex work and between trafficking for sex work and trafficking into other labour sectors. Further, they claim an indispensable role for sex workers alongside governments in the struggle against trafficking (GAATW 2018, 9). But Juno Mac and Molly Smith, both UK based sex worker activists chide the movement for this strategy as it suggests “that the current mode of anti-trafficking policy is broadly correct and merely—on occasion—misfires” (Mac and Smith 2018, 85). They convincingly argue why the anti-trafficking paradigm must be contested, root and branch. Katie Cruz similarly mobilises a Marxist feminist critique of the free-forced dichotomy of the anti-trafficking paradigm to map the continuum of unfreedom (Cruz 2018, 74, 84). I have also already shown how the anti-trafficking paradigm is deeply complicit with sex work exceptionalism such that prising it apart from its unintended consequences is impossible. I further argue that the current strategy of the sex workers’ movement also illustrates the highest cost yet—of anti-trafficking discourse, namely, how it prevents valuable research and strategic thinking on redistributive justice within the sex sector. Here, I interrogate and critique the movement’s theory of exploitation, regulation and internal redistribution, which it must prioritise going forward.

## Exploitation

In the desire to critique anti-trafficking discourse and draw a bright line between force and consent, one is hard-pressed to detect a theory of exploitation. Consider the following quote protesting the raid, rescue and rehabilitation strategy of neo-abolitionist groups in India:

As one child of a sex worker said, ‘I am so happy that our younger children are getting educated and have alternative ways by which they can earn their livelihood without necessarily coming into sex work… When a woman wants to give up sex work after her children are working is that not punarvasthi [rehabilitation]?’ (GAATW 2018, 23)

Battle weary from the trafficking debates, we are conditioned to read this statement as a rejection of neo-abolitionism’s focus on rehabilitation which is aimed at abolishing sex work. But from a redistributive standpoint, we should instead be asking if in fact the goal is to exit from sex work, on one’s terms, then does not removing the state from the sex sector, say by decriminalising sex work, go only half way towards realising this goal? We need a better understanding of how sex workers can protect their economic interests by earning more and improving their working conditions and bargaining power within sex work.

Similarly, Danielle, a sex worker from New Zealand offers a pithy critique of anti-trafficking discourse:

It means you ignore the ways in which you are being exploited, which are the same boring ways that anyone’s exploited under capitalism… The kind of exploitation that most of us are facing is the exploitation of working long hours, the uncertain pay, of management trying every trick they can to scam every dollar out of you that they can… It’s not the exploitation of being chained to a bed and raped for twelve hours straight… And in saying that that’s what we’re experiencing just invalidates when something bad does happen to you. And it makes it hard to recognise when bad things are happening when you’re always thinking, “Well at least I’m not, you know, at least I’m not in ‘Taken.’” (GAATW 2018, 20)

This extract highlights Chantal Thomas’ legitimation critique (Halley et al. 2006, 390), which is that focusing on the sensationalist cases of sex trafficking masks mundane exploitation in other sectors and in sex work itself as Danielle’s statement suggests. Why then are sex workers’ groups not studying the political economy of sex work enough to tell us more about the long working hours, uncertain pay and devious management techniques in New Zealand, a country that is routinely held up as a pioneer for decriminalising sex work? These reports also mention but do not elaborate on the oppressive bar rules in Thailand’s sex sector, alcohol consumption laws in South Africa, usury laws in Sangli, India and tenancy laws in London, Mumbai and South Africa where sex workers face the routine threat of eviction (Amnesty International 2016c, 4, 8, 11, 16, 22; AATW 2018, 58, 136, 169, 222). This is not to say that all sex workers’ groups are silent on exploitation. Thailand-based Empower has applied the ILO’s decent work parameters to its working conditions (Empower 2016) and the South African Sex Workers Education and Advocacy Taskforce (SWEAT) has drafted a Counter Sexual Exploitation in Sex Work Protocol (Yingwana et al. 2019, 84). But these initiatives are uncommon, amidst the flurry of reports from sex workers’ groups on the ills of anti-trafficking policy.

## Regulation

Sex workers have rightly rejected conventional forms of legalisation in favour of decriminalisation to get the state off their backs and avoid onerous regulatory burdens on what is an informal economy. The various reports above also assert that there is no proof that decriminalisation and legalisation increases or decreases trafficking or increases or decreases the size of the sex sector (International Committee on the Rights of Sex Workers in Europe 2016, 99; Amnesty International Explanatory Notes 2016b, 45; see also Bettio et al. 2017). While these claims are made to counter unsubstantiated and alarmist neo-abolitionist arguments that such policies expand the sector and increase sex trafficking, sex workers’ groups must rethink their assertions. Amnesty’s report has improved the policy vocabulary on sex work but remains geared towards formal state law, particularly criminal law. In reality, we already have considerable levels of de facto decriminalisation and therefore need a more nuanced assessment of precisely how formal rule changes to decriminalise or legalise sex work will in fact affect sex workers in relation to other laws, norms and governance structures that shape sex work. The results, as I argue elsewhere, can be counter-intuitive (Kotiswaran 2011).

In refining the regulatory vocabulary, we also need to appreciate informality in criminal justice systems from a comparative perspective. In developing countries, the criminal justice system is under-resourced, leading to a gap in enforcement but also perverse enforcement of the criminal law due to the rent-seeking practices of state officials. The size of the sex sector must also be considered for reworking a theory of regulation. To illustrate, the Swedish model is often held up to represent neo-abolitionism and the New Zealand model to represent decriminalisation. But both countries have around 3000 sex workers. Can these models realistically work in countries with larger sex worker populations like South Africa (153,000), Spain (45,000 to 300,000), Thailand (300,000), Mexico (600,000), or India (two to 10 million) (GAATW 2018)? The resultant gap in enforcement due to limited state resources results in self-regulation in these very jurisdictions—the self-regulatory boards in Kolkata, the Thantha Mukti Committee or dispute resolution committees in Sangli (GAATW 2018, 109), the role of gang members in preventing trafficking and resolving disputes in South Africa (id., 223) and the appointment by the Nicaraguan Supreme Court of sex workers as judicial mediators (Cabezas 2019, 48–49).

## Hierarchies Within the Sex Sector

Finally, reports on sex work and trafficking assume that sex workers comprise a discrete group whose common interests can be served if only we could achieve the right kind of reform through advocacy. The conflicting stakes that sex workers have vis-à-vis each other rarely come up except with migrant sex work given its link with the visible and polarising issue of immigration. Ava Caradonna of the x:talk project in the UK notes:

What is clear is that we have reached a historical moment, one in which the political significance of sex worker rights has finally started to gain momentum and traction. That is why it is crucial that we, as migrant sex workers, address some of the conflicts of interest between the aims and strategies of the sex workers’ movement as they are currently configured and the strategies and realities of migrant sex workers themselves. (Caradonna 2016, 25)

Caradonna claims that even sex workers’ main demand to recognise sex work as work is a double-edged sword for migrants as their right to work is not always guaranteed and that full decriminalisation would not help undocumented sex workers in the UK (id., 26). This leads to a splintering in positions amongst sex workers vis-à-vis anti-trafficking law. Caradonna claims that the sex workers’ movement cannot offer migrant sex workers much, while the rescue industry can offer access to legal aid, counselling, temporary housing, professional training, and possibly asylum (id., 28). Nicola Mai’s ethnography of migrant sex workers in Europe confirms the value of a trafficking visa (Mai 2018, 118). Thus the anti-trafficking complex hurts sex workers’ interests but can also benefit the most marginalised sex workers. Remarkably, the mainstream sex workers’ movement in France and Canada have linked their demands with those of migrant sex workers. However, we also need to consider the differentiated stakes of non-migrant sex workers as this queries the ethics of representation within the sex workers’ movement which at its current moment of strength needs to take stock of which sex worker voices are filtering through into the advocacy space, and at whose expense.

## The Postcolonial Politics of Anti-trafficking Discourse

In part one, I have shown how anti-trafficking law and policy entrench sex work exceptionalism despite the adoption by states of a secular Art 3 definition of trafficking in their domestic laws. In part two, I have shown how the efforts of the international sex workers’ movement to counter it by contesting the conflation of trafficking with voluntary sex work is not only bound to fail but is also a distraction from the urgent pursuit of a radical politics for redistribution within sex work. In particular, I have elaborated on how this focus has severely undermined the sex workers’ movement’s ability to produce a sophisticated theory of regulation to reduce levels of exploitation within sex work, one which is commensurate with the informality and heterogeneity of sex markets the world over. In this final part, I return to influential theories for the persistence of sex work exceptionalism in the global North and how it depends on legitimation through its international travels yet how global South countries are not always the passive recipients of sex work exceptionalism.

The sex panic accompanying the rise of the anti-trafficking complex in the West has long relied on transnational ‘others’, memorialised by New York Times journalist Nicholas Kristof’s expeditions into third world red-light areas in India and Cambodia to rescue sex slaves from brothels. Postcolonial feminists have castigated such anti-trafficking campaigns for reinforcing problematic colonial-era constructs of the third world victim “as thoroughly disempowered, brutalised and victimized” (Kapur 2005, 115) and infantilised or passive (Sanghera 2005, 13). Kamala Kempadoo has also criticised the neocolonialist exclusion by Western sex workers’ groups of third world sex workers from their struggles (Kempadoo 1998, 13).

But the relation between sex work exceptionalism in the global North and global South is deeper and warrants closer examination. Elizabeth Bernstein and Nicola Mai have recently explained the cause for sex work exceptionalism in the US and Europe. Bernstein has reworked the idea of the ‘sex panic’ and the ‘strange bedfellows’ thesis between radical feminists and evangelical Christians to theorise the sexual politics of anti-trafficking discourse in terms of carceral feminism, militarised humanitarianism and redemptive capitalism. Carceral feminism in her words, is a “cultural and political formation in which previous generations’ struggles for gender justice and sexual liberation are recast in terms of criminal justice” (Bernstein 2018, 21). Militarised humanitarianism refers to neo-colonial humanitarian interventions in the global South using undercover raid, rescue and rehabilitation missions on the part of Western anti-slavery crusaders to save third world women from third world men (id., 22). Redemptive capitalism meanwhile aims to solve the problem of sex trafficking rather than address poor labour conditions within post-Fordist working arrangements, but in the process extends its surveillance over sex work (id., 23, 147–154). Feminists and evangelists alike, Bernstein argues, subscribe to a “new politics of sex and gender” (id., 13), converge on criminal justice responses to social problems (id., 78) and support market-based agendas for gender freedom. She maps how such thinking finds expression and legitimation through charity tourism in Thailand where Western tourists are taken to trafficking ‘hotspots.’ Mai similarly uses the term ‘sexual humanitarianism’ to describe the global emergence of a neo-abolitionist epistemology that legitimises targeted forms of control and protection of social groups, defined as vulnerable in relation to their sexual orientation and behaviour (Mai 2018, vii). The export of the Swedish model he notes, is an example of moral gentrification by the global North (id., xi). If this alliance of social actors in the global North derives legitimacy from its international ventures, we need a robust account for what sustains or refracts anti-trafficking discourse abroad. We need to ask why despite the rapid expansion of the Swedish model, only one country which has adopted the Swedish model, namely, South Africa is in the global South. This requires us to take a closer look at a developing world context which may help us reimagine an alternate politics of sex worker organising. I draw on experiences in one such context, namely, India to trace the path that neo-abolitionism has taken there and why the fortunes of global governmentality in the global South produce quite unexpected outcomes for sex workers’ mobilisation. I consider India in particular because it continues to capture the imagination of the leading anti-modern day slavery crusaders of our times like Kevin Bales. The Global Slavery Index also estimates that India has the highest absolute numbers of modern slaves in the world, 18 million being the flow figure (Walk Free Foundation 2016) and eight million, the stock figure (Walk Free Foundation 2018). Significantly, India is also home to around a million sex workers (NACO 2016, 39) and remains the backdrop against which victim narratives of enslaved third world sex workers make it into anti-trafficking and neo-abolitionist discourse.

At the outset, feminist interventions on sex work and trafficking in India trace a path different to carceral feminism in the US. Given the strong materialist feminist tradition of the Indian women’s movement, feminists have been ambivalent about sex work and have not prioritised sex work or trafficking in their advocacy efforts (Kotiswaran 2019c, 393). Intra-feminist disagreements have resulted in policy paralysis rather than pitch-forked battles over law reform (unlike on say rape) (id.). Their abdication of this space has generated a vacuum, which has been occupied by neo-abolitionist NGOs comprising both radical feminist NGOs but also non-feminist conservative groups which seek to abolish sex work to restore the dignity of Indian women and children. These NGOs rely on militarised humanitarian interventions such as raids, rescue and rehabilitation, enabled by substantial international, especially USAID funding to counter trafficking. Since 2000, India alone has received 19 million USD a year (Gleason and Cockayne 2018, 8). Various state feminists in turn, support these NGOs, the most influential being the Ministry of Women and Child Development.

Sex workers meanwhile have been at the receiving end of the state’s brutal violence through the Immoral Traffic Prevention Act, 1956 or ITPA,^12^ India’s anti-sex work criminal law, which does not criminalise the sale of sex per se but criminalises activities necessary for sex work. However, sex workers have also been exposed to the state’s softer discursive power through HIV prevention projects. They have used HIV prevention resources to build a robust sex workers’ movement and push back against abolitionist proposals. The first such occasion arose in 2004 when the US State Department placed India in the Tier Two Watch list in its annual Trafficking in Persons (TIP) report, leading the Indian government to propose criminalising customers of sex workers. Sex workers protested before Parliament but also leveraged their centrality to HIV prevention efforts to argue that the Swedish model would undo the government’s own HIV prevention work. They mobilised the dissonance at the heart of the Indian state by pitting the Ministry of Health against the ministries of Home Affairs and Women and Child Development. Despite their success, however, they shied away from state-oriented reform, whether to repeal the ITPA or pursue litigation.

Unlike sex workers’ groups, from the 1990s, neo-abolitionist NGOs actively engaged with the state. They were repeat players before the courts when they used the liberal locus standi requirements of public interest litigation to secure the prosecution of traffickers and protection for victims. They collaborated outside the courtroom with the police, district magistrates, anti-human trafficking units and children’s welfare committees to conduct raid and rescue operations and draft standard operating procedures and manuals. Thus, they became the ‘expert’ go-to groups when the state decided to legislate on trafficking pursuant to India’s ratification of the Protocol. Further, after the 2012 rape and murder of Jyoti Pande, when the government appointed the Verma Committee to suggest rape law reform, these groups prevailed on the Committee to introduce an offence which conflated trafficking with voluntary sex work. Sex workers’ groups, with the help of feminist lawyers obtained a retraction from the Committee (Pawar 2013). The resultant anti-trafficking offence, Sect. 370 of the Indian Penal Code, 1860 (IPC) is modelled on the Protocol’s Art 3 definition.

This victorious pushback was however short-lived. A lawsuit filed by a neo-abolitionist NGO in 2004 came alive in 2015 and became the impetus for the government to propose the Trafficking of Persons (Prevention, Protection and Rehabilitation) Bill in 2016 (Government of India 2016). The 2018 version (‘Trafficking Bill’) was a highly draconian law which built out Sect. 370 into a substantial penal and welfare bureaucracy, using state surveillance to prevent trafficking. It further provided for the state and private NGOs to operate protective and rehabilitation homes for victims of trafficking; the Bill had a provision where a judge could discount a victim’s desire not to be sent to such a home, if the judge thought he/she had been coerced. The Bill was passed in July 2018 by the lower house of Parliament where it lapsed.

It would be easy to read the Bill’s passage as a show of strength for the neo-abolitionists who had the ear of the then Minister of Women and Child Development, Smt Maneka Gandhi. However, testament to the strength of sex worker organising were her repeated reassurances that the Bill would not affect voluntary sex work. In an op-ed (quoted at the start of this article), she even aligned the Bill’s objective with the interests of the sex workers’ movement. Neo-abolitionist commentators followed suit, by arguing that the Bill would pave the way for decriminalisation because its drafters were non-judgmental and provided victims a right to rehabilitation but did not mandate it (Bharadwaj 2018). A chimera of sex worker agency was permitted, that too to assert the right to rehabilitation, continuing in a long tradition of a complex middle-ground feminism wherein both state feminists and feminists in the women’s movement believe that given India’s high levels of poverty, we can only recognise the rights of sex workers but not the right to sex work (Kotiswaran 2019b, 11–12). Yet there is no better illustration of the seductive offer of a secular anti-trafficking law that apparently exempts voluntary sex work when in fact, the Trafficking Bill channelled the very spirit of the ITPA, did not repeal the ITPA and was on all counts, a draconian criminal law bound to adversely affect several vulnerable groups in addition to sex workers.

The sexual politics of anti-trafficking discourse in India resonates with developments in the West, particularly the international spread of militarised humanitarianism fuelled by USAID funding. However, underlying this is an indigenous paternalist approach to sex work that emerged post-independence when women leaders of the nationalist movement lobbied for the passage of the Suppression of Immoral Traffic Act 1956 (the predecessor of the ITPA). Rather than penalise sex workers, who they considered to be fallen women and victims of economic circumstance, law-makers prioritised rehabilitation by female police officers in state-run homes staffed by female social workers (De n.d., 29–30, 33). Conservative neo-abolitionists have today donned this paternalist mantle within a space abdicated by materialist feminists (rather than form coalitions with carceral feminists as they have in other contexts). Thus, India is not simply a playground for moral gentrification by international neo-abolitionists. Also, while states in the global South converge with international neo-abolitionist groups on carceral anti-trafficking agendas, the fortunes of what Akhil Gupta calls global governmentality (Gupta 2012, 239) are unpredictable. Thus, the Indian government has long resented the TIP ranking system, has recently protested the Global Estimates of Modern Slavery (GEMS) released by the Walk Free Foundation and the ILO (Debroy 2017) and reportedly denied visas to researchers from the Walk Free Foundation after the 2016 Global Slavery Index claimed that India had the world’s largest number of ‘modern slaves.’ Similarly, although international actors claim legislative success (Grono 2016), law reform is often triggered by domestic political opportunities like the 2012 rape case and the extensive use of public interest litigation by neo-abolitionist NGOs which led to the formulation of the Trafficking Bill.

Both Bernstein and Mai also explain carceral feminism, sexual humanitarianism and redemptive capitalism in terms of the rise of neoliberal penality alongside a withdrawal of the state’s welfare functions. The coherence of neoliberal penality has been challenged in the West (O’Malley 2016), and resonates even less in the Indian context given the trajectory of the developmental state, the decrepit criminal justice system inherited through colonial rule and a leaky welfare bureaucracy. Paradoxically, the inauguration of neoliberal economic policies in the 1990s alongside structural adjustment has meant not a reduction, but rather an increase in its welfare function. There has thus been less of a radical break with the 1970s and 1980s, when India pursued a socialist model of development and also enacted laws on bonded labour, contract labour and inter-state migrant work to prevent what is today understood as ‘trafficking.’ An activist Supreme Court even held that any labour paid below the minimum wage was ‘forced labour,’ reiterating it more recently in the context of the large-scale national rural employment guarantee scheme launched almost anachronistically after India introduced neoliberal economic policies in the early 1990s. Connections were however rarely made between this forced labour jurisprudence and trafficking; the latter was and continues to be understood as prostitution. Consequently, the labour movement has offered sex workers’ groups little support in countering the neo-abolitionist complex.

The expansion rather than retraction of the developmental state has however presented Indian workers, most of whom work in the informal economy, an opportunity to bargain for their rights outside of labour laws which cater to workers in the formal economy. In a study of social movement unionism in the cigarette and construction sectors in three Indian states, Rina Agarwala concludes that rather than fight flexible production structures and demand traditional work benefits such as minimum wages and job security, these workers demand state responsibility as voters, for meeting their social consumption and reproductive needs for education, housing and health care (Agarwala 2013, 15). The state is open to these demands while continuing to pursue neoliberal economic policies.

For years, sex workers have pursued a similar strategy. Rather than follow neo-abolitionist NGOs in pursuing legislative change, or litigating or working with the executive on the ground, sex workers instrumentalise the law as a ‘shield’ against the state’s attempts to abolish sex work, rather than as a ‘sword’ to further their workers’ rights. Their mobilisational efforts are fragile and contingent. They reshape themselves according to governmental categories as ‘day labourers’, ‘people living below the poverty line,’ demand inclusion in welfare programs as a ‘needful’ category, and demand land titles as landless people, as well as old age pension, night shelters and a safe working environment (Kotiswaran 2013, 541). Even when campaigning for workers’ rights, they align with rag pickers, street vendors, and scavengers in the ‘unorganised sector.’ Instead of mounting a constitutional challenge to the ITPA, they have lobbied provincial governments to add sex work to the occupation schedule of the central and state Ministries of Labour. The state has been responsive to such demands although it is hostile to appeals for decriminalisation, much less workers’ rights (id.). More recently, in the aftermath of the COVID-19 pandemic and associated lockdowns, sex workers’ groups approached state governments, the National Human Rights Commission and the Supreme Court (in an ongoing case) to demand dry rations and financial assistance on a monthly basis. The National Human Rights Commission issued a human rights advisory on the rights of women during the COVID-19 pandemic which recognised sex workers as informal workers who it stated should be registered for worker benefits and welfare measures even if they are unable to produce ration cards or citizenship documents (NHRC 2020) but this has since been modified to offer sex workers these benefits on “humanitarian grounds” instead.

Thus anti-trafficking discourse has been sustained in India by a different matrix of arrangements: by a middle-ground feminism that supposedly speaks to ‘India’ realities and through the unpredictable play off between international militarised humanitarianism and indigenous traditions of paternalist protectionism which leads to alliances between domestic and international neo-abolitionists, but is punctured by moments of nationalist pride and resistance to US imperialism and global governance. Further, the Indian state is thoroughly governmentalised, presenting both neo-abolitionists and sex work advocates with opportunities for influencing those in power. Neo-abolitionists have actively engaged with the executive, judiciary and legislature while sex work advocates have been on the defensive and warded off the Swedish model. Tragically, although India has embraced criminal, labour *and* development approaches to extreme exploitation, it is the criminal law approach of the Trafficking Bill that has gained ascendance. Sex workers meanwhile have become more visible, formed alliances with active social movements (e.g. transgender rights activists), effectuated de facto decriminalisation through informal arrangements with the police and resorted to novel self-regulation, while aligning with workers in the informal economy to secure their social reproduction needs through the logic of competitive populism.

## Conclusion

In conclusion, anti-trafficking discourse has morphed considerably over the past 20 years with the term ‘trafficking’ managing to hold various disparate carceral projects together and intensifying sex work exceptionalism rather than diffusing it. The international sex workers’ movement has defied sex work exceptionalism but at the cost of redistributive efforts within the sex sector. As the Protocol continues its international travels fuelled by neoabolitionist funding, it meets resistance in the global South where each country has its own deep histories of contestations over sex work and indigenous approaches to extreme exploitation. Allies remain few and far between; feminists are either guilty of commissioning a carceral approach as in the US or of the error of omission as in India. Labour movements are themselves embattled, and where they are not, sex workers remain the lumpen proletariat and therefore ignored. Strategic choices for future action are highly contextual but sex workers continue to find their strength in movements for structural reform such as Black Lives Matter, Occupy Wall Street, migrant solidarity, reforming drug policy and with other labour groups as this image of a May Day Rally in Kolkata in 2018 shows, where sex workers marched along with domestic workers, agricultural workers, embroidery workers, and local cigarette workers to demand that they be included as workers in the labour schedule of the federal government. It is this hope that animates sex workers’ struggles for the future.
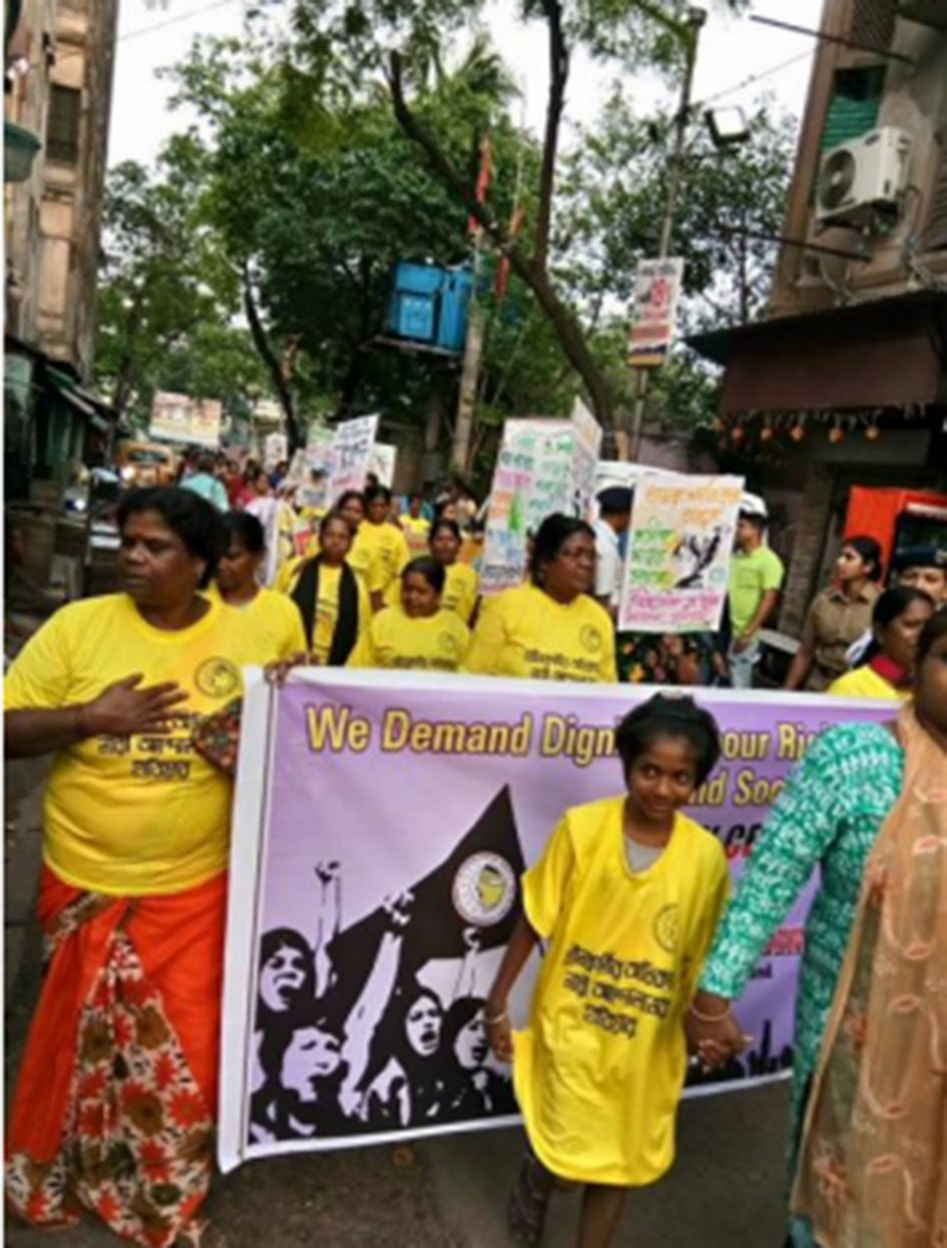


## Footnotes

1 Art 3 of the Protocol defines exploitation to “include, at a minimum, the exploitation of the prostitution of others or other forms of sexual exploitation, forced labour or services, slavery or practices similar to slavery, servitude or the removal of organs.”

2 Abolishing slavery and involuntary servitude.

3 https://www.ohchr.org/Documents/Issues/Trafficking/ConceptNoteAgenda20thAnniversaryPalermoProtocol.pdf.

4 Comments made during a webinar 29–30 June 2020, see: https://www.ohchr.org/EN/Issues/Trafficking/Pages/20-Years-After.aspx.

5 Art. 3 requires three elements to be proved for the trafficking of adults—an action element which includes transporting or movement or harboring; a means element which includes fraud, deception, coercion, abuse of power, abuse of a position of vulnerability and finally, the purpose of trafficking, namely, any one of the various forms of exploitation, including, slavery, practices similar to slavery, the exploitation of the prostitution of others, forced labour and so on.

6 Council of Europe 2005 Art 26; EU Directive 2011 Art 8.

7 Principle 7 states: Trafficked persons shall not be detained, charged or prosecuted for the illegality of their entry into or residence in countries of transit and destination, or for their involvement in unlawful activities to the extent that such involvement is a direct consequence of their situation as trafficked persons.

8 California Evidence Code 1965, Senate Bill No. 233.

9 Some clarification here about the four main policy approaches to sex work. These are: prohibition or complete criminalization where all aspects of sex work and actors, including sex workers, are criminalized; complete decriminalization where any special anti-sex work criminal law is repealed, leaving sex work to be regulated by other laws applicable to all citizens, including, the general criminal law; abolition or partial decriminalization where all aspects of and players in sex work, including customers, are criminalized although sex workers themselves are not; and legalization, where the form of regulation could vary, but where in addition to the repeal of the anti-sex work criminal law, special rules regulating sex work are put into place. (Halley et al. 2006).

10 [2010] (10) BCLR 1029 (LAC).

11 These include the World Health Organization, the Joint United Nations Programme on HIV/AIDS, United Nations Development Program, UN Women, the Office of the UN High Commissioner for Human Rights, the UN Special Rapporteur on the Right to Health, the UN Special Rapporteur on Violence Against Women, the Committee on Economic, Social and Cultural Rights, the Committee on Torture and the UN Human Rights Committee (Amnesty International 2016b, 27). Support is also found in the ILO Recommendation on HIV and the World of Work, the Report of the Expert Group Meeting for the Commission on the Status of Women, and several commissions on HIV and the Law (id.).

12 https://indiacode.nic.in/.

## References

[CR1] Agarwala Rina (2013). Informal Labour, Formal Politics and Dignified Discontent in India.

[CR2] Ahmed Aziza, Kotiswaran Prabha (2017). Addressing HIV/AIDS as the Intersection of Anti-Trafficking and Health Law and Policy. Revisiting the Law and Governance of Trafficking, Forced Labour and Modern Slavery.

[CR3] Amnesty International. 2016a. Policy on State Obligations to Respect, Protect and Fulfil the Human Rights of Sex Workers. Pol 30/4062/2016.

[CR4] Amnesty International. 2016b. Explanatory Note on Amnesty International’s Policy on State Obligations to Respect, Protect and Fulfil the Human Rights of Sex Workers. Index: POL 30/4063/2016.

[CR5] Amnesty International, 2016c Sex Workers at Risk: A Research Summary on Human Rights Abuses Against Sex Workers. Index: POL 40/4061**/**2016

[CR6] Andrijasevic Rutvica (2007). Beautiful Dead Bodies: Gender, Migration and Representation in Anti-Trafficking Campaigns. Feminist Review.

[CR7] Anti-Slavery International. 2016. International Cotton Campaign against Slavery. https://www.antislavery.org/take-action/campaigns/end-uzbek-cotton-crimes/. Accessed 24 November 2020.

[CR10] Austin, Rachel, and Amy Farrell. 2017. Human Trafficking and the Media in the United States. In *Oxford Research Encyclopedia Criminology and Criminal Justice*, ed. Nicole Rafter and Michelle Brown. USA: Oxford University Press.

[CR11] Bazelon, Emily. 2015. The Return of the Sex Wars. *New York Times*. 10 September.

[CR12] Bell Laurie (1987). Good girls/bad girls: sex trade workers and feminists face to face.

[CR14] Bernstein Elizabeth (2007). The Sexual Politics of the “New Abolitionism”. Differences.

[CR15] Bernstein Elizabeth (2018). Brokered Subjects: Sex Trafficking and the Politics of Freedom.

[CR16] Bettio Francesca, Giusta Marina Della, Tommaso Maria Laura Di (2017). Sex Work and Trafficking: Moving beyond Dichotomies. Feminist Economics.

[CR17] Bharadwaj, Abza. 2018. Anti-trafficking Bill: No, it does not take away sex workers’ rights. Here’s how. *Daily O,* 20 December. https://www.dailyo.in/user/15015/abza_bharadwaj. Accessed 20 January 2020.

[CR18] Blanchette, Thaddeus and Laura Murray. 2016. The power of putas: the Brazilian prostitutes’ movement in times of political reaction. In *Sex workers speak. Who listens?*, ed. P.G. Macioti and Giulia Garofalo Geymonat, 75–80. Open Democracy: Beyond Trafficking and Slavery. https://cdn-prod.opendemocracy.net/media/documents/BTS_Sex_Workers_Speak.pdf. Accessed 14 October 2020.

[CR19] Boecking Benedikt, Miller Kyle, Kennedy Emily, Dubrawski Artur (2019). Quantifying the Relationship between Large Public Events and Escort Advertising Behavior. Journal of Human Trafficking.

[CR20] Cabezas Amalia L (2019). Latin American and Caribbean Sex Workers: Gains and challenges in the movement. Anti-Trafficking Review.

[CR21] California Evidence Code 1965, Senate Bill No. 233, assented to by Governor July 30, 2019, http://leginfo.legislature.ca.gov/faces/billNavClient.xhtml?bill_id=201920200SB233. Accessed 14 October 2020.

[CR22] Caradonna, Ava. 2016. We speak but you don’t listen: migrant sex worker organising at the border. In *Sex workers speak. Who listens?* ed. P.G. Macioti and Giulia Garofalo Geymonat, 25–29, Open Democracy: Beyond Trafficking and Slavery. https://cdn-prod.opendemocracy.net/media/documents/BTS_Sex_Workers_Speak.pdf. Accessed 14 October 2020.

[CR23] Chuang Janie C (2010). Rescuing Trafficking from Ideological Capture: Prostitution Reform and Anti-Trafficking Law and Policy. U. Penn L. Rev..

[CR24] Clean Clothes Campaign. 2020. Deadly Indian factory fire again shows need for preventive safety measures and justice for workers. 17 February. https://cleanclothes.org/news/2020/deadly-indian-factory-fire-again-shows-need-for-preventive-safety-measures-and-justice-for-workers. Accessed 14 October 2020.

[CR25] Cohen Amy J, Gruber Aya, Halley Janet E, Kotiswaran Prabha, Rebouché Rachel, Shamir Hila (2019). Governance Feminism in New York’s Human Trafficking Intervention Courts. Governance Feminism: Notes from the Field.

[CR26] Cossman Brenda, Rehman Javaid, Shahid Ayesha, Foster Steve (2019). MeToo, Sex Wars 2.0 and the Power of Law. The Asian Yearbook of Human Rights and Humanitarian Law.

[CR27] Council of Europe Convention on Action Against Trafficking in Human Beings (Warsaw, 5 May 2005), C.E.T.S. No. 197 (entered into force 1 February 2008).

[CR28] Council of the European Union. 2011. 2011/36/EU (2011) Council Directive 2011/36/EU of 5 April 2011 on Preventing and Combating Trafficking in Human Beings and Protecting its Victims.

[CR29] Cruz Katie (2018). Beyond Liberalism: Marxist Feminism, Migrant Sex Work, and Labour Unfreedom. Feminist Legal Studies.

[CR30] Davida Grupo (2015). Trafficking as a Floating Signifier: The View From Brazil. Anti-Trafficking Review.

[CR31] De, Rohit. *Husna Bai’s Profession: Sex, Work and Freedom in the Indian Constitution* (copy on file with author).

[CR32] De Stone, Roshan and David Suber. 2019. The Domestic Workers Resisting Slavery in Lebanon, 16 September. https://newint.org/features/2019/09/16/domestic-workers-resisting-slavery-lebanon. Accessed 14 October 2020.

[CR33] Debroy, Bibek. 2017. International Labour Organisation’s Omissions. *The Indian Express*, 30 November. https://indianexpress.com/article/opinion/columns/international-labour-organisation-modern-slavery-forced-marriage-4960954/. Accessed 20 January 2020.

[CR34] Doezema Jo (2005). Now you see her, now you don’t: Sex workers at the UN Trafficking Protocol Negotiations. Social & Legal Studies.

[CR35] Doezema Jo, Kempadoo Kamala (1998). Forced to Choose: Beyond the Voluntary v Forced Prostitution Dichotomy. Global Sex Workers Rights, Resistance, and Redefinition.

[CR36] Empower Foundation. 2012. Hit and Run: The impact of anti trafficking policy and practice on Sex Workers’ Human Rights in Thailand. https://www.nswp.org/sites/nswp.org/files/Hit%20and%20Run%20%20RATSW%20Eng%20online.pdf. Accessed 14 October, 2020.

[CR37] Empower Foundation. 2016. Moving Toward Decent Work. Sex Worker Community Research: Decent Work and Exploitation in Thailand. https://www.nswp.org/sites/nswp.org/files/Moving%20Toward%20Decent%20Sex%20Work%20Summary%2C%20EMPOWER%20-%20April%202016.pdf. Accessed 20 January 2020.

[CR38] Farrell Amy, Delateur Monica J, Owens Colleen, Fahy Stephanie (2016). The Prosecution of State-Level Human Trafficking Cases in the United States. Anti-Trafficking Review.

[CR39] Gallagher AT (2016). Editorial: The Problems and Prospects of Trafficking Prosecutions: Ending impunity and securing justice. Anti-Trafficking Review.

[CR40] Gandhi, Maneka. 2018. Why I pushed for passage of the anti-trafficking bill. *Times of India*, 30 July. https://timesofindia.indiatimes.com/india/why-i-pushed-for-passage-of-the-anti-trafficking-bill/articleshow/65190751.cms. Accessed 20 January 2020.

[CR41] Geist Darren (2016). Amnesty International's Empty Promises: Decriminalization, Prostituted Women, and Sex Trafficking. Dignity A Journal on Sexual Exploitation and Violence.

[CR42] Gleason, Dr Kelly A and Dr James Cockayne. 2018. Official Development Assistance and SDG Target 8.7: Measuring aid to address forced labour, modern slavery, human trafficking and child labour. Delta 8.7 and United Nations University Centre for Policy Research.

[CR43] Global Alliance Against Traffic in Women. 2007. Collateral Damage: The Impact of Anti-Trafficking Measures on Human Rights Around the World, ed. Michael Dottridge. https://gaatw.org/resources/publications/908-collateral-damage-the-impact-of-anti-trafficking-measures-on-human-rights-around-the-world. Accessed 14 October 2020.

[CR8] Global Alliance Against Traffic in Women. 2016. *Anti-Trafficking Review*, Special Issue on Representations of Trafficking, September, ed. Rutvica Andrijasevic and Nicola Mai, Issue No. 7, pp. 1–199.

[CR44] Global Alliance Against Traffic in Women. 2018. Sex Workers Organising for Change: Self-representation, community mobilization, and working conditions. https://www.gaatw.org/publications/SWorganising/SWorganising-complete-web.pdf. Accessed 14 October 2020.

[CR9] Global Alliance Against Traffic in Women. 2019. *Anti-Trafficking Review*, Special Issue on Sex Work, ed. Annalee Lepp and Borislav Gerasimov, Issue No. 12, pp. 1–204.

[CR90] Government of India. 2016. Ministry of Women and Child Development, Trafficking of Persons (Prevention, Protection and Rehabilitation) Bill. 2016. https://wcd.nic.in/sites/default/files/Draft%20Trafficking%20of%20persons%20Bill%202016.pdf

[CR45] Grono, N. (2016). Measuring slavery encourages governments to do better. *Thomson Reuters Foundation News*. 16 July. http://news.trust.org/item/20160716164413-tig2w. Accessed 2 March 2020.

[CR46] Gupta Akhil (2012). Red Tape Bureaucracy, Structural Violence, and Poverty in India.

[CR47] Halley Janet, Kotiswaran Prabha, Shamir Hila, Thomas Chantal (2006). From the International to the Local in Feminist Legal Responses to Rape, Prostitution/Sex Work, and Sex Trafficking: Four Studies in Contemporary Governance Feminism. Harvard Journal of Law and Gender.

[CR48] Halliday Terence, Levi Michael, Reuter Peter (2019). Anti-Money Laundering: An Inquiry into a Disciplinary Transnational Legal Order Transnational Legal Order. UC Irvine Journal of International, Transnational, and Comparative Law.

[CR49] Hill Annie (2016). How to Stage a Raid: Police, media and the master narrative of trafficking. Anti-Trafficking Review.

[CR50] Hodal, Kate, Chris Kelly and Felicity Lawrence. 2014. Revealed: Asian slave labour producing prawns for supermarkets in US, UK. *The Guardian,* 10 June. www.theguardian.com/globaldevelopment/2014/jun/10/supermarket-prawns-thailand-produced-slavelabour. Accessed 14 October 2020.

[CR51] Human Rights Watch. 2015. *Precious Metal, Cheap Labor, Child Labor and Corporate Responsibility in Ghana’s Artisanal Gold Mines*. https://www.hrw.org/report/2015/06/10/precious-metal-cheap-labor/child-labor-and-corporate-responsibility-ghanas. Accessed 14 October 2020.

[CR52] International Committee on the Rights of Sex Workers in Europe. 2016. Amnesty International: adopt the proposed policy on sex work International Committee on the Rights of Sex Workers in Europe. In *Sex workers speak. Who listens?* ed. P.G. Macioti and Giulia Garofalo Geymonat, 94–101. Open Democracy: Beyond Trafficking and Slavery. https://cdn-prod.opendemocracy.net/media/documents/BTS_Sex_Workers_Speak.pdf. Accessed 14 October 2020.

[CR53] International Labour Organisation (2009). Fighting Forced Labour: The Example of Brazil.

[CR200] Immoral Traffic (Prevention) Act. 1956 as amended in 1986. https://www.indiacode.nic.in.

[CR54] International Labour Office, Walk Free Foundation and International Organization for Migration. 2017. *Global Estimates of Modern Slavery: Forced Labour and Forced Marriage*. Geneva.

[CR55] Kapur Ratna (2005). Erotic Justice Law and the New Politics of Postcolonialism.

[CR56] Kelly, Annie. 2018. From nail bars to car washes: how big is the UK's slavery problem? *The Guardian*, 18 October. https://www.theguardian.com/global-development/2018/oct/18/nail-bars-car-washes-uk-slavery-problem-anti-slavery-day. Accessed 14 October 2020.

[CR57] Kempadoo Kamala, Kempadoo Kamala, Doezema Jo (1998). Introduction: Globalizing Sex Workers’ Rights. Global Sex Workers: Rights, Resistance, and Redefinition.

[CR66] Kotiswaran Prabha (2011). Dangerous Sex, Invisible Labor: Sex Work and the Law in India.

[CR67] Kotiswaran Prabha (2013). Sword or Shield? The Role of the Law in the Indian Sex Workers’ Movement Interventions. International Journal of Postcolonial Studies.

[CR58] Kotiswaran Prabha. 2018. Criminal Law as Sledgehammer: The Paternalist Politics of India’s 2018 Trafficking Bill, 9 July https://www.opendemocracy.net/en/beyond-trafficking-and-slavery/criminal-law-as-sledgehammer-paternalist-politics-of-india-s-2018-tr/. Accessed 2 March 2020.

[CR68] Kotiswaran Prabha (2019). Trafficking: A Development Approach. Current Legal Problems..

[CR69] Kotiswaran Prabha (2019). Has the Dial Moved on the Indian Sex Work Debate?. Economic & Political Weekly.

[CR70] Kotiswaran Prabha, Halley Janet, Kotiswaran Prabha, Rebouché Rachel, Shamir Hila (2019). Governance Feminism’s Others: Sex Workers and India’s Rape Law Reforms. Governance Feminism: Notes from the Field.

[CR59] Lloyd P, Simmons BA, Halliday TC, Shaffer G (2015). Framing and Transnational Legal Organization: The Case of Human Trafficking. Transnational Legal Orders.

[CR60] Mac Juno, Smith Molly (2018). Revolting Prostitutes The Fight for Sex Workers’ Rights.

[CR61] Mai Nicola (2018). Mobile Orientations: An Intimate Autoethnography of Migration, Sex Work, and Humanitarian Borders.

[CR62] National AIDS Control Organisation, Ministry of Health and Family Welfare, Government of India (2016). Mid-Term Appraisal of National AIDS Control Programme Phase IV.

[CR63] National Human Rights Commission. 2020. *Human Rights Advisory on Rights of Women in the Context of COVID-19*. https://nhrc.nic.in/media/press-release/nhrc-issues-advisories-rights-women-context-covid-19-concerned-ministries-and. Accessed 14 October 2020.

[CR89] Network of Sex Work Projects. 2020. NSWP Strategic Plan 2016-2020. https://www.nswp.org/resource/nswpstrategic-plan-2016-2020. Accessed 11 December 2020.

[CR64] O'Malley, Pat. 2016. *Neoliberalism, Crime and Criminal Justice*. Research Paper No. 16/10, Sydney Law School. 10.2139/ssrn.2729627. Accessed 14 October 2020.

[CR65] Pawar, Yogesh. 2013. Voluntary Sex Work Is Legal. *Daily News and Analysis,* 24 March. https://www.dnaindia.com/mumbai/report-voluntary-sex-work-is-legal-1814995. Accessed 14 October 2020.

[CR71] Richardson, B. 2016. Still Slaving Over Sugar*.* In *E-Book on Forced Labour in the Global Economy*, ed. G. LeBaron and N. Howard, 74–77. Open Democracy: Beyond Trafficking and Slavery Series. https://www.dnaindia.com/mumbai/report-voluntary-sex-work-is-legal-1814995. Accessed 17 March 2017.

[CR72] Sanford Rachealle, Martínez Daniel E, Weitzer Ronald (2016). Framing Human Trafficking: A Content Analysis of Recent U.S. Newspaper Articles. Journal of Human Trafficking.

[CR73] Sanghera Jyoti, Sanghera Kamala Kempadoo Jyoti, Pattanaik Bandana (2005). Unpacking the Trafficking Discourse. Trafficking and Prostitution Reconsidered: New Perspectives on Migration, Sex work, and Human Rights.

[CR74] Sangram. 2018. *Raided: How Anti-trafficking Strategies Increase Sex Workers’ Vulnerability to Exploitative Practices*. Sangli. https://sangram.org/upload/news/newsPdf/raided-e-book-4.pdf. Accessed 14 October 2020.

[CR75] Sardina, Cristine. 2019. *Marketing mass hysteria: anti-trafficking awareness campaigns go rogue*. Open Democracy: Beyond Trafficking and Slavery, 20 June. https://www.opendemocracy.net/en/beyond-trafficking-and-slavery/marketing-mass-hysteria-anti-trafficking-awareness-campaigns-go-rogue/. Accessed 14 October 2020.

[CR76] Scoular Jane (2015). The Subject of Prostitution: Sex Work, Law and Social Theory.

[CR77] Skrivankova, Klara. 2010. *Between Decent Work and Forced Labour: Examining the Continuum of Exploitation*. Joseph Rowntree Foundation Programme Paper. York: Joseph Rowntree Foundation.

[CR79] UN Office of the High Commissioner for Human Rights (OHCHR). 2002. Recommended Principles and Guidelines on Human Rights and Human Trafficking. E/2002/68/Add.1

[CR78] UN Office of the High Commissioner for Human Rights (OHCHR). 2020. The importance of implementing the non-punishment provision: the obligation to protect victims, Special Rapporteur on trafficking in persons, especially women and children.

[CR80] United Nations. 2000a. Protocol to Prevent, Suppress and Punish Trafficking in Persons, Especially Women and Children, Supplementing the United Nations Convention Against Transnational Organized Crime. 15 November 2000, in force 25 December 2003. 2237 UNTS 319. New York.

[CR81] United Nations. 2000b. Convention Against Transnational Organized Crime. 15 November 2000, in force 29 September 2003. 2225 UNTS 209. New York.

[CR82] United Nations Office on Drugs and Crime. 2014. Global Report on Trafficking in Persons. U.N. Pub. Sales No. E.14.V.10.

[CR83] United Nations Office on Drugs and Crime. 2016. Global Report on Trafficking in Persons. 48, U.N. Pub. Sales No. E.16. IV. 6.

[CR84] Vollbehr, W. 2016. Sex Workers against Human Trafficking: Strategies and challenges of sex worker-led organizations in the fight against human trafficking, Master Thesis, Vrije Universiteit Amsterdam.

[CR85] Walk Free Foundation. 2016. *Global Slavery Index* Australia.

[CR100] Walk Free Foundation. 2018. *Global Slavery Index* Australia.

[CR86] Wijers M (2015). Purity, Victimhood and Agency: Fifteen years of the UN Trafficking Protocol. Anti-Trafficking Review.

[CR87] Yingwana N, Walker R, Etchart A (2019). Sex Work, Migration, and Human Trafficking in South Africa: From polarised arguments to potential partnerships. Anti-Trafficking Review.

[CR88] x: talk. 2010. Human Rights, Sex Work and the Challenge of Trafficking: Human rights impact assessment of anti-trafficking policy in the UK, October. http://www.xtalkproject.net/wp-content/uploads/2010/12/reportfinal1.pdf. Accessed 11 December 2020.

